# The swimming test is effective for evaluating spasticity after contusive spinal cord injury

**DOI:** 10.1371/journal.pone.0171937

**Published:** 2017-02-09

**Authors:** Youngjae Ryu, Toru Ogata, Motoshi Nagao, Taku Kitamura, Kazuhito Morioka, Yoshinori Ichihara, Toru Doi, Yasuhiro Sawada, Masami Akai, Ryohei Nishimura, Naoki Fujita

**Affiliations:** 1 Department of Veterinary Surgery, Graduate School of Agricultural and Life Sciences, The University of Tokyo, Tokyo, Japan; 2 Department of Rehabilitation for the Movement Functions, Research Institute, National Rehabilitation Center, Saitama, Japan; 3 Department of Neurosurgery, Brain and Spinal Injury Center, University of California, San Francisco, California, United States of America; 4 Graduate School, International University of Health and Welfare, Tokyo, Japan; University of Toronto, CANADA

## Abstract

Spasticity is a frequent chronic complication in individuals with spinal cord injury (SCI). However, the severity of spasticity varies in patients with SCI. Therefore, an evaluation method is needed to determine the severity of spasticity. We used a contusive SCI model that is suitable for clinical translation. In this study, we examined the feasibility of the swimming test and an EMG for evaluating spasticity in a contusive SCI rat model. Sprague-Dawley rats received an injury at the 8th thoracic vertebra. Swimming tests were performed 3 to 6 weeks after SCI induction. We placed the SCI rats into spasticity-strong or spasticity-weak groups based on the frequency of spastic behavior during the swimming test. Subsequently, we recorded the Hoffman reflex (H-reflex) and examined the immunoreactivity of serotonin (5-HT) and its receptor (5-HT_2A_) in the spinal tissues of the SCI rats. The spasticity-strong group had significantly decreased rate-dependent depression of the H-reflex compared to the spasticity-weak group. The area of 5-HT_2A_ receptor immunoreactivity was significantly increased in the spasticity-strong group. Thus, both electrophysiological and histological evaluations indicate that the spasticity-strong group presented with a more severe upper motor neuron syndrome. We also observed the groups in their cages for 20 hours. Our results suggest that the swimming test provides an accurate evaluation of spasticity in this contusive SCI model. We believe that the swimming test is an effective method for evaluating spastic behaviors and developing treatments targeting spasticity after SCI.

## Introduction

Spasticity is a common complication after traumatic spinal cord injury (SCI). Traumatic disconnection between the upper and lower motor neurons is known to result in hyperexcitability of spinal circuits and subsequent spastic symptoms, including muscle spasms, muscular hypertonia, and clonus [1–5]. Spasticity frequently impairs voluntary motor control, and its severity varies among individuals with SCI. Skold et al. (1999) reported that approximately 65–80% of patients with SCI present with muscle spasticity, whereas others have reported that 50–60% of patients complain of spastic symptoms that interfere with voluntary motion [2, 6, 7]. Although a series of pharmacological treatments, such as GABA agonists, have been used clinically, there are still many patients with unsolved spastic symptoms. To improve upon and develop new spasticity-targeted therapeutic interventions, the underlying mechanisms of spasticity and voluntary motor control need to be elucidated.

The pathophysiology of spasticity has been well studied in rodent models mainly using complete spinal cord transection models, wherein supraspinal input is completely disconnected beyond the lesion [8, 9]. These studies have provided insights into spasticity mechanisms, including exaggerated spinal reflexes, alternations in synapses, and changes in the expression of motor neuron receptors after SCI [10–16]. However, inflammatory tissue reactions and the spared neural network, which exerts a critical influence on the spinal circuitry after injury, differ greatly between the complete spinalized model and the contusive model with incomplete paralysis [17–19]. Considering that most patients with spasticity have a traumatic contusive spinal cord injury, using an incomplete contusive rodent model is more appropriate for clinical translation than the use of a complete model. Although several studies on spasticity using contusive injury animal models have shown increased muscle resistance and spinal hyperreflexia after SCI [20–22], individual variation in spastic symptoms remains an obstacle for the detailed analysis of molecular mechanisms underlying spasticity. Therefore, methods need to be developed to evaluate spasticity in a contusive SCI model to enable further quantification and analysis of spasticity.

The swimming test is a well-established approach for evaluating behavior after SCI [23, 24]. Gonzenbach et al. (2010) reported muscle spasms in an incomplete transection SCI rat model during swimming. However, to our knowledge, there are no criteria for evaluating spastic behavior in a contusive SCI model. We therefore attempted to use a swimming test for the screening and quantification of spasticity, as well as to determining its validity in a contusive SCI rat model. In particular, we sought to determine whether a swimming test can be effectively used to quantify spastic behaviors and to determine the severity of spasticity in a contusive SCI rat model. These findings are expected to help in the design of future experiments and representative models used to improve our understanding of the mechanisms underlying the development of spasticity following SCI.

## Materials and methods

### Experimental animals, surgery, and post-operative care

Ten-week-old female Sprague-Dawley rats (n = 60, 200–300 g, Charles River Japan) were used in this experiment. Fifty rats received the contusive injury, and 10 were used as uninjured controls. The rats were anesthetized using pentobarbital sodium (50 mg/kg, intraperitoneal [I.P.], Kyoritsu Seiyaku Corporation, Tokyo, Japan) and received a 250-kilodyne (kd) contusive injury from an Infinite Horizon impactor device (Precision Systems and Instrumentation, VA, USA) at the 8th thoracic level. After surgery, the injured rats were treated with a single shot of antibiotic (Baytril, 5 mg/kg, SC, Bayer). Manual bladder expression was conducted twice per day until the injured rats spontaneously urinated. The housing room was under a 12-hour/12-hour light/dark cycle and the temperature was maintained at 23°C. Food and water were supplied *ad libitum*. Uninjured rats were used as controls. All animal experiments were approved by the ethical committee of the National Rehabilitation Center for Persons with Disabilities.

### Swimming tests

**Fig 1:**
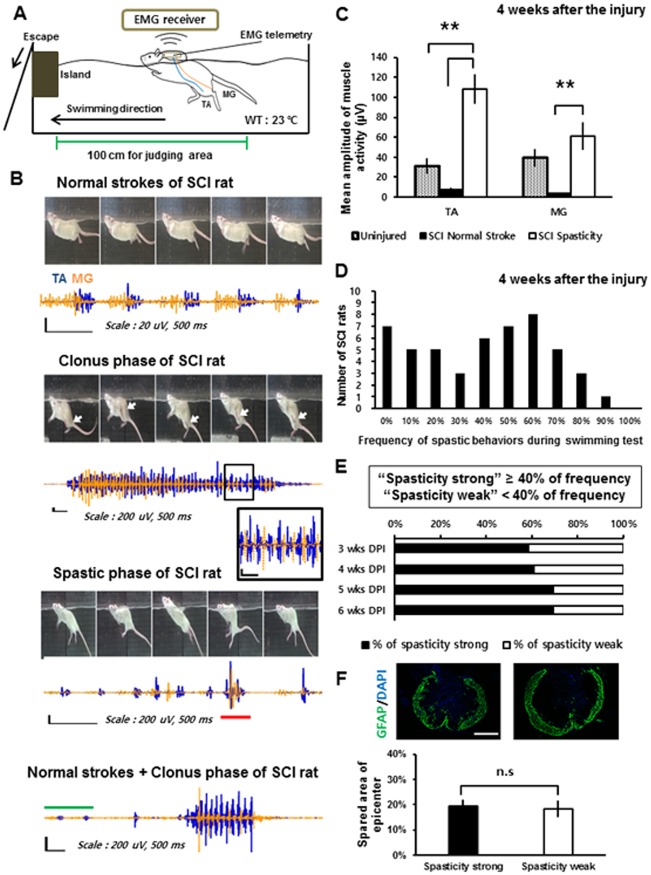
Types and occurrence frequencies of spastic behaviors during the swimming test. (A) Configuration of the EMG recording device during the swimming test. (B) Sequential captured images and corresponding recorded muscle activity of the left TA (blue) and the left MG (orange) muscle during the swimming test showing each type of spasticity (the clonus phase and spastic phase). The black box represents a magnified image of the recorded EMG. The red bar on the recorded EMG of the spastic phase indicates co-contraction of the TA and MG muscles. The green bar on the bottom image corresponds to the normal reciprocal stroke phase. White arrows indicate the location of the hind paw of the rat during the clonus phase. Note that all EMG figures were normalized to the peak amplitudes of TA muscle activity. (C) Mean amplitude of muscle activity during spastic behaviors (SCI spasticity; black bar) compared with the mean amplitude of muscle activity during normal reciprocal strokes (SCI normal stroke; white bar) of SCI rats and uninjured rats (Uninjured; hatched bar) at 4 weeks after SCI. (D) The number of the SCI rats with each occurrence frequency of spastic behaviors during swimming test at 4weeks after the injury. (E) Percentage of the spasticity-strong rats from 3 to 6 weeks after SCI. Injured rats were classified as “spasticity-strong” if they showed the occurrence frequency over 40% during the swimming test. (F) Average percentage of the spared area of the epicenter of the injury measured by the GFAP (+) area after SCI in the spasticity-strong group and -weak group. **: p < 0.01 Abbreviations: WT, water temperature; TA, tibialis anterior; MG, medial gastrocnemius; n.s, not significant.

Swimming tests were performed in a rectangular Plexiglas chamber (150 × 14.5 × 40 cm) filled with tap water to a depth of approximately 20–23 cm. The water temperature was maintained at 23°C, which is considered to be an optimal temperature for the rat swimming test [25]. The swimming direction of the rats was controlled from the left to the right side of the chamber using a ramp and an island attached to the left side of the chamber (Fig 1A). Assessment of swimming was conducted at a point 100 cm from the starting and ending points. Swimming from the start point to the end point was regarded as a single run of the swimming test. We performed the swimming test over 5 runs 3 weeks after SCI and over 10 runs each 4, 5, and 6 weeks after SCI. If the SCI rats had at least one clonus phase or a spastic phase in a single run during the test, they were classified as a “spasticity-positive” rat (*for criteria for clonus and spastic phases*, *see the* Results *section*). Even if the animals had multiple or mixed phases during a run, the run was considered to be a “spasticity-positive” run because it was difficult to independently determine the specific episodes. If both spastic and clonus phases were observed during a run, the run was classified as a clonus phase run. Importantly, behaviors related to defecating or urinating during swimming were excluded. All swimming tests were videotaped using a Sony Handycam HDR-CX700 camera at 60 frames per second.

### EMG telemetry implantation and recording

We implanted an EMG telemetry device (model: F40-EET, DSI/Receiver model: RPC-1, DSI, MN, USA) to obtain EMG data while the rats moved freely. The EMG telemetry device was inserted between 3 and 4 weeks after the SCI. The device was implanted at the same age in the uninjured animals. The rats (n = 15) were anesthetized using pentobarbital sodium (50 mg/kg, I.P.). After anesthesia, the body of the EMG telemetry device was inserted subcutaneously into the neck region and fixed using sutures. The EMG wires were led via subcutaneous injection and implanted intramuscularly into the left tibial anterialis (TA) muscle, which acts as an ankle flexor, and the left medial gastrocnemius (MG) muscle, which has the opposite action as an ankle extensor. The wires were tightly fixed using 6–0 nylon sutures. EMG recording was performed at least 3 days after surgery. After EMG implantation, the rats were treated with a single shot of antibiotic (Baytril, 5 mg/kg, SC). The EMG signal (sampled at 1,000 Hz) was digitized and filtered using a high-pass filter (30 Hz). EMG recordings were synchronized with a video recording using a light cue trigger. Spike2 software (CED, Cambridge, England) was used for the EMG recording and analysis. All EMG recordings were obtained in an electromagnetic-shielded room.

### Twenty-hour cage observation

Cage observations were conducted 4 weeks after SCI from 16:00 on the first day to 12:00 on the following day. Each rat (n = 10) with an implanted EMG telemetry device was individually housed in a large plastic cage (40 × 25 × 18 cm) under dim light. Video was captured using a web camera during simultaneous EMG recording. EMG recording was synchronized to the video recording using a light-cue trigger. For quantitative evaluation, we counted the numbers of clonus and spasm episodes during the 20-hour cage observations, which were confirmed with using EMG recordings (see Results section). Behaviors during defecation or urination were excluded. The same rats that were used for the cage observations were used for the EMG recordings on a treadmill (Robomedica, Inc., CA, USA) with body weight support 4 weeks after SCI to examine muscle activity during voluntary motion. EMG recordings during 10 cycles of walking gaits on a treadmill were obtained at a rate of 7.5 m/minute.

### Hoffmann reflex (H-reflex) recording

Under chloral hydrate anesthesia (2.5 g/kg, Sigma) delivered via intraperitoneal injection, we measured the Hoffmann reflex (H-reflex). After anesthesia, the left or right hindlimb was incised to expose the distal tibial nerve. The Bipolar cuff was hooked onto the distal tibial nerve at ankle level and a pair of recording electrodes was inserted into the same side of the plantar muscle of hind limb subcutaneously. The distance between the recording electrodes was 3 mm and the tip of electrodes was exposed to a length of about 1.5 mm. A ground electrode was attached to the surface of the tail. To determine the rate-dependent depression (RDD) of the H reflex, the tibial nerve was stimulated at 0.2, 0.5, 1, 2, and 5 Hz using a stimulator (1–2 V of intensity, Nihon Kohden, Japan). The EMG signal (sampled at 5,000 Hz) was passed to an amplifier (NEC Biotop 6R12, Nihon Kohden) and band-pass filtered (5–3,000 Hz). At each stimulation frequency, 20 serial stimulations were performed and the first 5 waves were discarded. The amplitudes of the M wave and the H wave were calculated by averaging 15 waves of each waveform measured using peak-to-peak values. The rate-dependent changes at each stimulation frequency were calculated as a percent of the response at 0.2 Hz. Spike2 (CED) was used for signal analysis.

### Tissue processing and immunohistochemistry

Six weeks after SCI, the rats were deeply anesthetized using sodium pentobarbital, perfused with PBS, and fixed using a 4% PFA solution delivered transcardially. The cervical region and the lumbar region (C1–C3 of the cervical enlargement and L4–L6 of the lumbar enlargement, respectively), and the epicenter of the SCI were removed and post-fixed in a 4% PFA solution for an additional 24 hours at 4°C. The tissues were cut transversely into 20-μm-thick sections on a cryostat (Leica CM3050S, Leica Microsystems). The tissue sections were incubated with diluted primary antibodies (goat-anti choline acetyltransferase, 1:100, Millipore; rabbit-anti serotonin 2A receptor, 1:200, Calbiochem; rabbit-anti NeuN, 1:500, Millipore; goat-anti serotonin, 1:500, Abcam; mouse-anti glial fibrillary acidic protein [GFAP], 1:500, Millipore) overnight. The next day, the sections were incubated with fluorescent secondary antibodies (Alexa Fluor 488 and 568 for each species-of primary antibody, 1:200, Life Technologies) and DAPI (1:1,000, Sigma) for 2 hours. All images were acquired using a BZ-9000 HS All-in-one Fluorescence Microscope (Keyence BZ-9000).

### Quantification and data analyses

The area of immunoreactivity (IR) was measured using Image J software (National Institutes of Health, USA). Serotonin 2A receptor (5-HT_2A_) levels were measured according to the methods described by Kong et al. (2011). In brief, we set thresholds for and binarized each image. Next, we calculated the 5-HT_2A_ receptor-labeled area corresponding to the ChAT (choline acetyltransferase)-labeled areas of the soma and proximal dendrites of the spinal motor neurons located in the ventral horn. Quantification of the area of serotonin (5-HT) staining was limited to the ventral horn of the spinal cord. Images of the ventral horn obtained at 20× magnification were analyzed for 5-HT immunoreactivity. Five sections (each section was separated by over 300 μm) were used for image analyses of the 5-HT_2A_ receptor and 5-HT fibers.

The spared area of the epicenter of the injury was calculated by measuring the GFAP(+) area in each spinal section outline area. EMG recordings were obtained and analyzed using MATLAB software (Mathworks). EMGs obtained over 1 second during spastic behaviors were used to determine the mean amplitude of muscle activity. Video analyses and motion capturing were performed using Vegas Pro software (Sony).

### Statistical analyses

We used SPSS software (IBM SPSS, Inc.) for all statistical analyses. All error bars are standard errors [SEs]. Statistical significance was set at *p* < 0.05.

## Results

### Spastic behaviors during swimming: clonus phase and spastic phase

After the mid-thoracic SCI, the injured rats were almost entirely dependent on their forelimbs during swimming. However, they occasionally used their hindlimbs in normal reciprocal strokes but with a slower cycle (< 2 Hz) compared to uninjured rats (4–5 Hz). During the normal strokes, the injured rats had clear reciprocal muscular activations in their hindlimbs, although some portions of the EMG pattern were overlapping (Fig 1B). We defined normal and reciprocal activation of the hindlimbs as the “normal stroke phase” for EMG comparisons. After at least 3 week of SCI, the injured rats had typical symptoms of spasticity during swimming. These symptoms were referred to as the “clonus phase” or the “spastic phase”. The clonus phase presented as rapid (5–8 Hz) jerking movements of either one or both legs during swimming (S1 Video). During this phase, simultaneously recorded EMGs showed reciprocal, high intensity bursts of TA and MG muscle activity (Fig 1B and 1C). It is notable that the clonus phase was often observed immediately after the rats stretched or contracted their hindlimbs. The spastic phase typically presented as a ventro-flexed trunk posture with stretched hind limbs during swimming (Fig 1B and S2 Video). A common feature of the spastic phase was the extended hindlimb posture, which was represented by co-contraction patterns of high bursts of the TA and MG muscle activity. The spastic behaviors, which were divided into the clonus phase and the spastic phase, were observed either simultaneously or independently within a single swimming run and were easily verified during the swimming test. Given that some of the common spastic symptoms observed after SCI include hyperreflexia, clonus, hypertonus of the muscles, and muscular spasm in both human patients and animal models [4, 26], we postulated that the clonus phase and the spastic phase observed during swimming represent the spastic symptoms of contusive SCI rats. The mean amplitude of muscle activity during the spastic phase in SCI rats was significantly higher than that during the normal stroke phase of both SCI and uninjured rats (*n* = 4 for uninjured rats, *n* = 5 for SCI rats, one-way ANOVA with Tukey’s *post-hoc* test; Fig 1C). In summary, the symptoms of spasticity in the contusive SCI rat model were observed during the swimming test.

### The swimming test can be used to classify SCI rats according to frequency of spastic behaviors

In the swimming experiment, we calculated the frequency of spastic behavior. The histogram of the occurrence frequency indicates the existence of two groups based on spastic behavior (*n* = 50 SCI rats; Fig 1D). Because the groups were apparently divided based on 40% frequency, we used a cut-off of 40% (i.e., 4 or more “spasticity-positive” runs among the 10 swimming runs) as the criterion for grouping SCI rats into the “spasticity-strong group.” The other SCI rats were grouped into the “spasticity-weak group”.

Three weeks after SCI, over 50% of the SCI rats were placed in the spasticity-strong group. However, some of the SCI rats in the spasticity-weak group were subsequently classified in the spasticity-strong group 4 weeks after SCI. The percentage of rats in the spasticity-strong group reached a plateau 4 to 5 weeks after SCI (*n* = 36 tested SCI rats, Fig 1E). The spared tissue area of the epicenter of the injured site, which was measured by staining for GFAP 6 weeks after SCI, was not significantly different between the two groups (*n* = 5 per group, Student’s *t*-test; Fig 1F). These results indicate that the SCI rats can be grouped according to severity based on the occurrence frequency of spastic behaviors, including the clonus phase and the spastic phase, during the swimming test.

### Spasticity during the swimming test was consistent and reproducible

**Fig 2:**
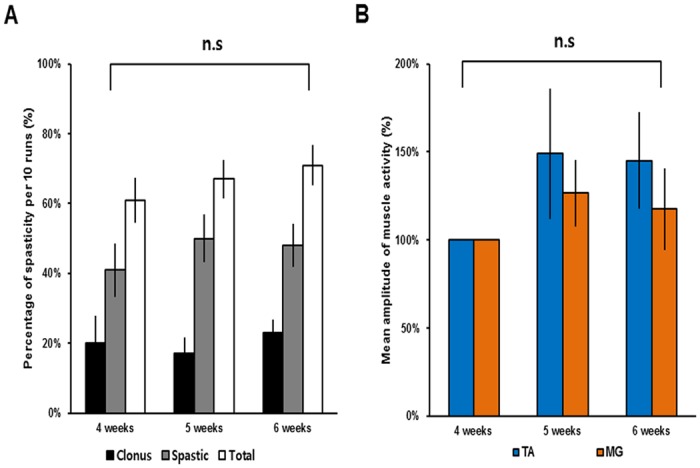
The occurrence frequencies of spastic behaviors and corresponding amplitudes of muscle activity during the test period. (A) The occurrence frequency and percentage of each type of spasticity (clonus, black bars; spastic, grey bars; total, white bars) observed in the spasticity-strong group during the swimming tests from 4 to 6 weeks after SCI. (B) The mean amplitude of muscle activity of spastic behaviors in the spasticity-strong group during the swimming test from 4 to 6 weeks after SCI recorded from the left TA muscle (blue) and the left MG muscle (orange). Mean amplitudes of muscle activity at 4 weeks were considered to be 100%. Abbreviations: TA, tibialis anterior; MG, medial gastrocnemius; n.s, not significant.

To determine whether the occurrence of spastic behaviors and the amplitude of muscle activity of each rat change over time, we performed the swimming tests weekly from 4 to 6 weeks after SCI. In the spasticity-strong group, the occurrence frequency of clonus and spastic phases during the 10 swimming runs was not significantly different from 3 weeks to 6 weeks after SCI (*n* = 10, one-way ANOVA with Tukey’s *post-hoc* test; Fig 2A). Furthermore, the mean amplitude of muscle activity during spasticity did not differ significantly between test periods in the spasticity-strong group (*n* = 6, Kruskal-Wallis H-test; Fig 2B). These results suggest that the occurrence frequency of spastic behaviors and the amplitude of muscle activity tend to be consistent during the time course, at least over our observation period.

### The spasticity-strong group had more hyperexcitability of the spinal reflex circuit

**Fig 3:**
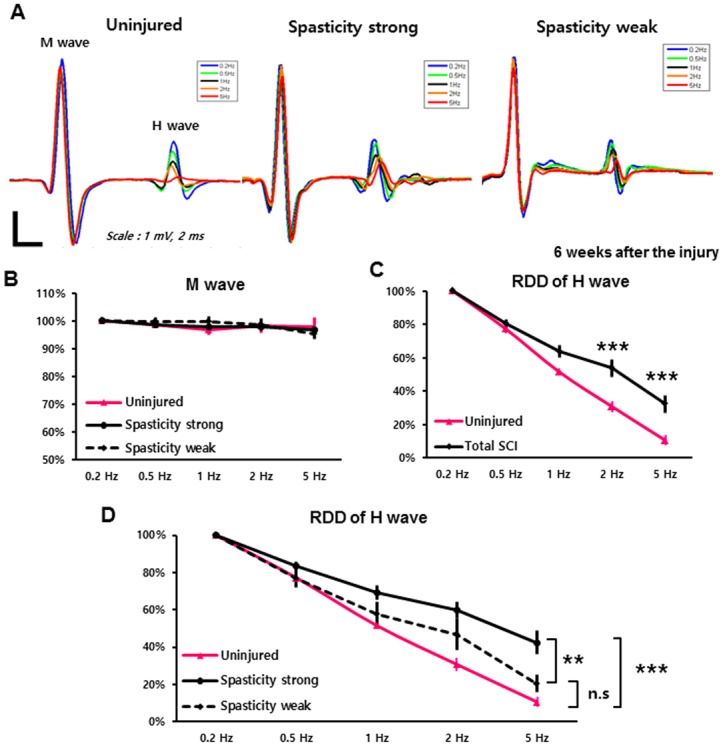
Rate-dependent depression (RDD) of the H-wave in the SCI groups, reflecting excitability of the spinal reflex. (A) Representative Hoffmann (H) reflex waveforms of uninjured, spasticity-strong, and -weak groups were shown. Different colors indicate different stimulation frequency: 0.2 Hz (blue), 0.5 Hz (Green), 1 Hz (Black), 2 Hz (orange), and 5 Hz (red). (B) RDD of the M-wave. The mean amplitude at 0.2-Hz stimulation was set to the baseline amplitude as 100%. Uninjured (pink), spasticity-strong group (solid line), -weak group (dashed line). (C) RDD of H-wave in the uninjured (pink) and total SCI group (black; includes spasticity-strong and -weak rats). (D) Comparison of the RDD values of the H-wave between groups. The RDD of the spasticity-strong group (solid black line), -weak group (dashed line), and uninjured rats (pink line). **: p < 0.01, ***: p ≤ 0.001.

To examine differences in the hyperexcitability of the monosynaptic lumbar spinal circuit between the spasticity-strong and spasticity-weak groups, the RDD of the H-reflex, which is classically used for evaluating spasticity, was obtained 6 weeks after SCI (Fig 3A). In control animals, the RDD of the M-wave was not changed by the stimulation frequency applied and was not significantly different between the uninjured group, the spasticity-strong group, and the spasticity-weak groups (*n* = 7–8 per group, two-way ANOVA with Bonferroni’s *post-hoc* correction; Fig 3B). The RDD of the H-wave for all SCI rats, including both spasticity-strong and spasticity-weak groups, was significantly less depressed than that of uninjured rats at stimulation frequencies of 2 Hz and 5 Hz, respectively (*n* = 7 for uninjured rats, *n* = 15 for SCI rats, two-way ANOVA with Bonferroni’s *post-hoc* correction; Fig 3C). The RDD of the spasticity-strong group was significantly more decreased than that of the spasticity-weak group at the stimulation frequency of 5 Hz. Furthermore, only the spasticity-strong group had statistically significant differences at stimulation frequencies of 1 Hz, 2 Hz, and 5 Hz compared to the uninjured group (n = 7–8 per group, two-way ANOVA with Bonferroni’s *post-hoc* correction; Fig 3D). These results suggest that the spasticity-strong group of SCI rats had more hyperexcitability of the lumbar circuit than the spasticity-weak group, which is consistent with the results of the behavioral screening performed during the swimming test.

### The spasticity-strong group has more up-regulated serotonin receptor expression in spinal motor neurons

**Fig 4:**
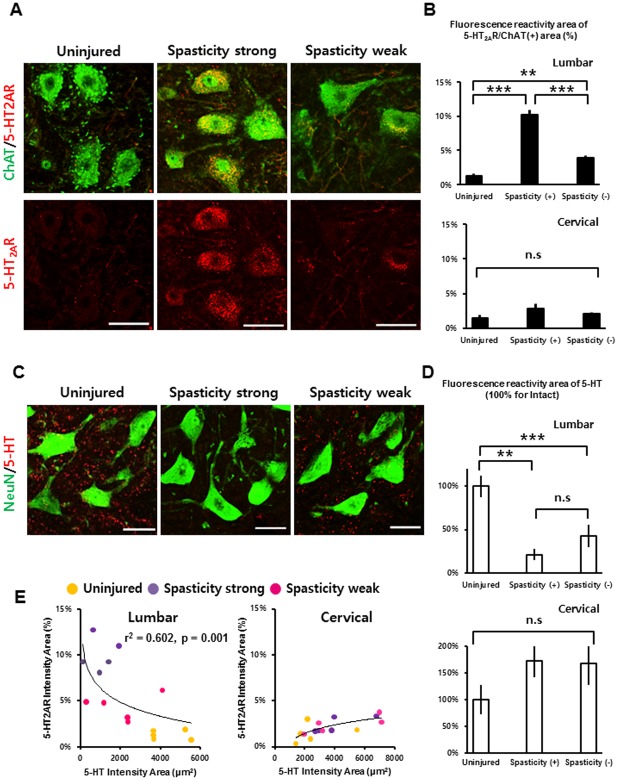
Immunohistochemistry and quantification of 5-HT and its receptor in SCI groups and uninjured rats. (A) Robust up-regulation of the 5-HT_2A_ receptor at the lumbar motor neurons of the spasticity-strong group compared with the -weak group and uninjured group (scale bars: 50 μm). (B) Quantification of the 5-HT_2A_ receptor-positive (+) area within spinal motor neurons labeled as ChAT positive (+) at both the cervical region and the lumbar region. (C) The 5-HT fibers were almost completely lost after 250-kd contusive SCI, but were more reduced at the spasticity-strong group (scale bars: 50 μm). (D) Quantification of the area of 5-HT fibers at both the cervical and the lumbar ventral horn region. Values were normalized considering the value for uninjured rats as 100%. (E) Plots of the quantification of 5-HT_2A_ receptor expression and 5-HT fibers at the cervical and lumbar region, respectively in uninjured (yellow), spasticity-strong group (purple), and -weak group (pink). **: p < 0.01, ***: p < 0.001 Abbreviations: 5-HT, 5-Hydroxytryptophan; ChAT, Choline acetyltransferase; n.s, not significant.

To determine other factors that might influence the variation between the spasticity groups, we examined serotonin (5-HT) fibers and 5-HT_2A_ receptors in both pre (cervical) and lower (lumbar) regions based on a report by Kong et al. (2011), who showed that expression of the serotonin 2A receptor was robustly increased after spinal cord transection in a rat model. Consistent with previous results, the IR of the 5-HT_2A_ receptor was greatly upregulated in the region below the epicenter of the spinal cord after SCI (Fig 4A). Furthermore, expression of the 5-HT_2A_ receptor in the lumbar motor neurons of the spasticity-strong group was significantly more upregulated compared to that of the spasticity-weak group (*n* = 5–6 per group, one-way ANOVA with Bonferroni’s *post-hoc* correction; Fig 4B). Conversely, the positive area of the 5-HT fibers in the ventral horn of the lumbar spinal cord was significantly reduced after SCI when compared to that of the uninjured rats. The 5-HT-positive area in the spasticity-strong group was also decreased when compared to that of the spasticity-weak group. However, this difference was not statistically significant (*n* = 5–6 per group, one-way ANOVA with Bonferroni’s *post-hoc* correction; Fig 4C and 4D). The IR areas of 5-HT_2A_ receptors and 5-HT fibers in the ventral horn of the cervical spinal cord did not show any significant differences between groups (*n* = 5–6 per group, one-way ANOVA with Bonferroni’s *post-hoc* correction; Fig 4C and 4D). Plots of reserved 5-HT fibers and expression patterns of the 5-HT_2A_ receptor in each animal suggest the presence of a strong relationship between the reserved 5-HT fiber area and 5-HT_2A_ receptor density in the lumbar spinal cord (r^2^ = 0.602, p = 0.001, logarithmic regression analysis; Fig 4E). The plot in Fig 4E also shows that uninjured rats have high 5-HT/5-HT_2A_R ratios, while spasticity-strong rats have low 5-HT/5-HT_2A_R ratios and spasticity-weak rats have intermediate ratios. These results indicate that the altered 5-HT/5-HT_2A_R ratio in the lumbar spinal cord may correlate with the frequency of spastic behaviors observed in the swimming test.

### Types and occurrence of spastic behaviors during the 20-hour observation period confirm the results of the swimming test

**Fig 5:**
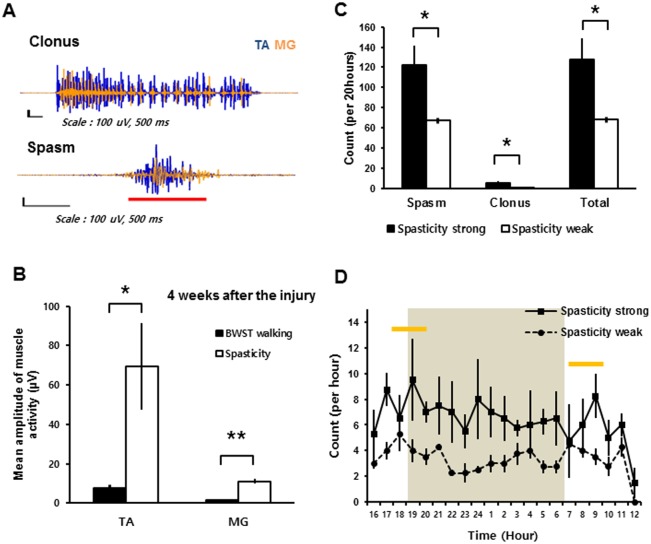
EMG results and occurrence frequencies of spastic behaviors during the 20-hour cage observation. (A) Recorded EMG results for the clonus and spasm in SCI rat during 20-hour cage observations obtained from the left TA muscle (blue) and the left MG muscle (orange). The red bar indicates co-contraction of the TA and MG muscle activities. Note that all EMG figures were normalized to the peak amplitudes of TA muscle activity. (B) Mean amplitude of muscle activity during spastic behaviors compared with the mean amplitude of muscle activity during 10 gait cycles on BWST 4 weeks after SCI. (C) The occurrence of each type of spastic behaviors during 20-hour cage observations in the spasticity-strong group (solid bars) and -weak group (open bars) at 4 weeks after SCI. (D) The occurrence of spastic behaviors in the spasticity-strong group (solid line) and -weak group (dashed line) shown by each recorded hour. The shaded area shows the default dark time (18:00 to 6:00) of the housing room. The yellow bars indicate the peak time points for the counted spastic behaviors. *: p < 0.05, **: p < 0.01 Abbreviations: TA, tibialis anterior; MG, medial gastrocnemius; BWST, body weight supported Treadmill.

To examine the correlation between spastic symptoms observed in the swimming test (Fig 1) and spastic behaviors under free-moving conditions on the ground, we conducted EMG recordings of the same SCI rats, which were tested and grouped using the swimming test, over 20 hours. The recordings were synchronized with video capture during normal behavior in a housing cage. Four weeks after SCI, the SCI rats had an episodic EMG pattern corresponding to a clonus or spasm episode in a cage situation (Fig 5A). Clonus during cage observations occurred episodically and was spontaneously accompanied by long-term (over 3 seconds based on the EMG recordings) reciprocal bursts of TA and MG muscle activity and abnormal motions, such as whirling of the tail (S3 Video). Spasms during the cage observations also occurred spontaneously with hyper-stretched limbs or hyper-contracted limbs, even when the rats were apparently sleeping or resting (S4 Video). The EMG patterns of the spasms during cage observations were represented by simultaneous and extensive bursts of TA and MG muscle activities. Since the muscle activities obtained during continuous gaits in the injured rats could not be assessed under normal cage conditions, we obtained a baseline amplitude for the gait on the treadmill. The mean amplitude of spastic muscle activity during the cage observations was significantly higher than that observed over 10 gait cycles on a body weight-supported treadmill in the same rats (*n* = 5, Student’s *t*-test; Fig 5B). The spasticity-strong group, which was assigned based on the results of the swimming test, had significantly more spasm and clonus episodes than the spasticity-weak group during the 20-hour observation period (*n* = 5 per group, Student’s *t*-test; Fig 5C). Although we also observed some specific types of spasms, such as extensor (MG)-related spasms or flexor (TA)-related spasms, these were not analyzed. Uninjured rats did not have any spasm or clonus episodes during the 20-hour observation period (*n* = 3). The average counted number of spasm and clonus episodes peaked in the early morning (08:00–09:00) and early evening (17:00–19:00) (*n* = 5 per group; Fig 5D), which is consistent with other reports regarding the spastic symptoms of rats and humans [25, 27]. These results indicate that the spasticity-strong group of SCI rats had more severe spasticity symptom than the spasticity-weak group during cage observation as well as during the swimming test.

## Discussion

We observed spastic behaviors during a swimming test in contusive SCI rats. The spastic behavior consisted of clonus and spastic phases. These behaviors were easily reproducible and detectable, and could be readily quantified based on occurrence frequency. In addition, the swimming test could discriminate SCI rats with different severities of spasticity. We used this test to classify the rats into the spasticity-strong group and the spasticity-weak group based on the occurrence frequency of the spastic behaviors. We confirmed the feasibility of this grouping using the H-reflex test, immunohistochemistry for the serotonin receptor, and the 20-hour cage observations. All measures consistently showed that the spasticity-strong group, which was assigned based on the swimming test, had more severe changes related to upper motor neuron syndrome compared to the spasticity-weak group. Furthermore, the percentage of SCI rats that were placed in the spasticity-strong group and the time course of spasticity after SCI are largely consistent with the results of a previous study using an incomplete transection rat model [25]. Therefore, the swimming test results reflect the severity of upper motor neuron syndrome, at least to some extent.

While we observed variations in severity among the spasticity-strong and spasticity-weak groups in the swimming test, we assume that our division into the two groups is reliable based on the results of multiple other modalities. Considering that incomplete SCI results in various degrees of spasticity (2, 6, 7), it is important to obtain homogenous experimental groups for SCI research. We thus suggest that the swimming test is a useful screening tool for incomplete SCI models.

We tested the hyperexcitability of the lumbar spinal circuit using the H-reflex test, which is a standard method of measuring hyperreflexia in both humans and animal models [15, 17, 28, 29]. We found that the spasticity-strong group had lower RDDs in the H-reflex, as well as increased expression of the 5-HT_2A_ receptor in the lumbar spinal motor neurons compared with the spasticity-weak group. Overexpression of the 5-HT receptor causes supersensitivity to serotonin, which is related to spasticity [30]. Therefore, we expected that such supersensitivity to serotonin, caused by the overexpression of serotonin receptors, may be the primary reason for the reduced RDD in the spasticity-strong group.

Supersensitivity of serotonin and up-regulation of serotonin receptors below the injury site after SCI have previously been reported to be related to the hyperexcitability of spinal motor neurons and consequent spasticity [13, 14, 16, 31–33]. It is known that several types of 5-HT_2_ receptors, such as 5-HT_2A_, 5-HT_2B_, and 5-HT_2C_, are closely related to the hyperexcitability of motor neurons after SCI [14]. However, immunohistochemical studies have clearly shown robust elevation of the 5-HT_2A_ receptor after SCI and reported the upregulation of mRNA for this receptor [34, 35]. Therefore, we selected the 5-HT_2A_ receptor as a representative serotonin receptor that is mainly expressed in spinal motor neurons for the present immunohistochemistry analysis [34, 36]. Our results indicate a correlation between the degree of serotonin supersensitivity and the severity of spasticity given the same severity of SCI.

During the 20-hour cage observations, we noted some spasticity-related behaviors in the “spasticity-weak” rats. Nevertheless, the total occurrence of spasm and clonus episodes was significantly lower in this group than in the spasticity-strong group. Therefore, our results suggest that the severity of spasticity in a contusive SCI rat model may be reflected by the occurrence frequency of spastic behaviors.

Our assessment using behavioral features may not directly reflect velocity-dependent muscle rigidity, which is the classical criterion for spasticity [37]. It should be noted that there are other established assays that use ankle-rotation evoked EMG responses (21, 22). Nevertheless, according to our EMG and H-reflex results, the behavioral patterns of the SCI rats during the swimming test correspond well to patterns of spastic symptoms observed in human patients with respect to 1) muscle spasms, 2) clonus, 3) co-contraction of the muscles, and 4) hypertonia and hyperreflexia. Therefore, the swimming test is able to show direct manifestations of clinically relevant symptoms of spasticity and may be useful for subsequent behavioral assessments. This test provides an advantage in prospective design by defining homogenous samples.

Our study has several limitations. First, the correlation of spastic behavior frequency during the swimming test and the H reflex test was not perfectly matched. Despite this limitation, we believe that the swimming test is useful for screening incomplete SCI rats to obtain more homogenous experimental groups. Second, spastic behaviors during the swimming test may not be able to be used to discriminate between voluntary attempts and involuntary movements. However, we found that those behaviors were unique, which allowed us to distinguish between the spasticity-strong and spasticity-weak groups. Furthermore, the behavioral assessment has the advantages of ease and repeatability for longitudinal experiments.

In summary, our results show that quantification and screening of spasticity in contusive SCI rats is possible by measuring the occurrence frequency of spastic behaviors during a swimming test. Our results may help to discriminate spasticity-weak rats from spasticity-strong rats after SCI. Taken together, our findings suggest that the swimming test is an effective method for evaluating symptoms of spasticity and developing treatments targeting spasticity after SCI.

## Supporting information

S1 Video Clonus phases during swimming test. (MP4)

S2 Video Spastic phases during swimming test. (MP4)

S3 Video Clonus during 20 hour cage observation. (MP4)

S4 Video Spasms during 20 hour cage observation. (MP4)
